# Flexible Actuator Based on Conductive PAM Hydrogel Electrodes with Enhanced Water Retention Capacity and Conductivity

**DOI:** 10.3390/mi13111951

**Published:** 2022-11-11

**Authors:** Yang Hong, Zening Lin, Yun Yang, Tao Jiang, Jianzhong Shang, Zirong Luo

**Affiliations:** College of Intelligence Science and Technology, National University of Defense Technology, Changsha 410073, China

**Keywords:** flexible actuator, conductive hydrogels, water retention, electronic conductor, conductivity, dielectric elastomer

## Abstract

Conductive polyacrylamide (PAM) hydrogels with salts that act as electrolytes have been used as transparent electrodes with high elasticity in flexible electronic devices. Different types and contents of raw materials will affect their performance in all aspects. We tried to introduce highly hydratable salts into PAM hydrogels to improve their water retention capacity. Different salts can improve the water retention capacity of PAM hydrogels to a certain extent. In particular, PAM hydrogels containing higher concentrations of lithium chloride (LiCl) and calcium chloride (CaCl_2_) showed an extremely strong water retention capacity and could retain about 90% and more than 98% of the initial water in the experimental environment at a temperature of 25 °C and a relative humidity of 60% RH, respectively. In addition, we conducted electrical conductivity tests on these PAM hydrogels with different salts. The PAM hydrogels containing LiCl also show outstanding conductivity, and the highest conductivity value can reach up to about 8 S/m. However, the PAM hydrogels containing CaCl_2_, which also performed well in terms of their water retention capacity, were relatively common in terms of their electrical conductivity. On this basis, we attempted to introduce single-walled carbon nanotubes (SWCNTs), multi-walled carbon nanotubes (MWCNTs), and graphene (GO) electronic conductors to enhance the electrical conductivity of the PAM hydrogels containing LiCl. The conductivity of the PAM hydrogels containing LiCl was improved to a certain extent after the addition of these electronic conductors. The highest electrical conductivity was about 10 S/m after we added the SWCNTs. This experimental result indicates that these electronic conductors can indeed enhance the electrical conductivity of PAM hydrogels to a certain extent. After a maximum of 5000 repeated tensile tests, the conductive hydrogel samples could still maintain their original morphological characteristics and conductivity. This means that these conductive hydrogel samples have a certain degree of system reliability. We made the PAM conductive hydrogels with high water retention and good conductivity properties into thin electrodes and applied them to an electric response flexible actuator with dielectric elastomer as the functional material. This flexible actuator can achieve a maximum area strain of 18% under an external voltage of 10 kV. This new composite hydrogels with high water retention and excellent conductivity properties will enable more possibilities for the application of hydrogels.

## 1. Introduction

In recent years, people have become interested in stretchable electronic products, thus significantly driving the demand for stretchable flexible conductive materials [1]. Stretchable and flexible conductive materials are crucial in many modern applications [2,3,4,5,6,7], such as flexible actuators, flexible sensors, multifunctional logic switches, and biomedical devices. At present, gel-based luminescent conductive materials have been widely used in biosensors and bioelectronics [8]. The biosensor that was improved by Zafar et al. [9] has ultra-fast response and high sensitivity properties, and it can be used for accurate and rapid biomedical detection. Some flexible electronic devices can be used as biosensors to detect the various kinds of target DNA and proteins such as the spike protein of COVID-19 [10]. Moreover, based on their tunable mechanical properties and excellent bio-compatibility, some flexible materials have been significantly applied in the biomedical field [11]. Currently, the most common flexible conductive materials are electronic conductors [12,13], such as carbon-based greases, liquid metals, and conductive particle/polymer composites. These electronic conductors generally cannot balance having a high transparency, stretchability, and good electrical conductivity [14,15]. Undoubtedly, these shortcomings limit their application value. In contrast, some ionic conductors such as conductive hydrogels have been found to meet these property requirements. These ionic conductors are highly stretchable with an elongation at break of up to 407% [16]. They are also highly transparent and have better shape adaptability than most electronic conductors do that are, themselves, colored [17,18]. In addition to the external properties, the inherent good electrical conductivity of the ion gels is the greatest strength supporting their wide application [19,20]. In 2013, Keplinger et al. [21] first published an application study on transparent ionic conductive hydrogels, making the application of conductive hydrogels possible. Thereafter, the research on conductive hydrogels has rapidly generated high-impact results. In their research, PAM conductive hydrogels with sodium chloride (NaCl) as the electrolyte exhibit excellent properties of high transparency, high stretchability, and ionic conductivity. The water in the hydrogels is used for the ionization of NaCl into sodium ions (Na^+^) and chloride ions (Cl^−^), which function as conductive ions. The conductive hydrogels have been practically applied to electrodes for transparent speakers. In addition, Jiang et al. [22] also developed a one-step strategy for the preparation of ionic gel, which gets rid of the complex preparation process that is involved in most existing electronic skins. Based on this method, they have prepared a smart e-skin with excellent ion transport and discriminability properties. This smart e-skin showed six stimulating responses to pressure, touch, temperature, humidity, magnetic force, and even astringency. This work is of great value, and it proposes a simple, efficient, controllable, and sustainable approach toward a low-carbon, versatile, and scalable e-skin design and structure–performance development.

Conductive gels are generally divided into conductive hydrogels with salts that act as electrolytes and ionic-liquid conductive gels [23]. Although ionic-liquid conductive gels do not suffer from dehydration failure, their electrical conductivity is relatively lower than that of conductive hydrogels [17]. Ionic liquids also interfere with some gel polymerization reactions, so the types of polymers that can be used to synthesize ionic liquid conductive gels are very limited [24]. In contrast, conductive hydrogels are generally easier to synthesize and easier to improve. Currently, conductive hydrogels mainly use various salts that act as the source of conductive ions. With the help of the ions that are generated by the dissolution of the salts in the water, a conductive network can be formed inside the conductive hydrogels. The type and concentration of conductive ions are the main factors that affect the conductivity of the conductive hydrogels [25]. In addition to conductivity, the water retention of conductive hydrogels during prolonged use should also be considered. The change of the moisture content directly affects the concentration of the conductive ions, which in turn affects the conductivity of the conductive hydrogels [26]. In summary, electrical conductivity and water retention capacity are the two most important issues in the research and use of conductive hydrogels. These properties directly affect the value of the conductive hydrogels in practical applications.

In this work, we explored the water retention effect of adding three salts to PAM hydrogels. Through an experimental comparison, it has been determined that LiCl and CaCl_2_ have outstanding effects on improving the water retention capacity of PAM hydrogels. Furthermore, we conducted electrical conductivity tests on PAM hydrogels containing different salts. In the experiment, the PAM hydrogels with a higher concentration of LiCl also showed outstanding advantages in electrical conductivity, but the effect of CaCl_2_ was general in enhancing the electrical conductivity. Based on the results of the water retention and electrical conductivity experiments, we identified the dominant position of LiCl in enhancing the comprehensive properties of the PAM hydrogels. In addition, we found the PAM hydrogels containing LiCl also show outstanding advantages in terms of conductivity. Subsequently, we introduced SWCNTs, multi-walled carbon nanotubes (MWCNTs), and graphene (GO) electron conductors to enhance the conductivity of the PAM hydrogels containing LiCl. However, the internal principles involved in these experimental phenomena still need to be further explored. Furthermore, we performed tensile tests on the conductive hydrogel samples to demonstrate the system’s reliability. Based on this, using PAM conductive hydrogels with high water retention and good conductivity, we designed and manufactured a flexible actuator with them to act as the flexible electrode material and carried out the actuation experiment of the flexible actuator under an external electric field excitation condition. The improved new composite PAM ionic conductive hydrogels will greatly expand the application scenarios of hydrogels.

## 2. Materials and Methods

### 2.1. Materials

The hydrogels were synthesized using acrylamide (AM; Sigma, A8887, Product of Switzerland) to act as the monomers, N, N-methylenebisacrylamide (MBAA; Sigma, 146072) as crosslinkers, ammonium persulfate (AP; Sigma, 248614) to act as the photo initiator, and N, N, N’, N’-tetramethylethylenediamine (TEMED; Sigma, T9281) to act as the crosslinking accelerator. The salts used to donate the ions in the PAM hydrogels were lithium chloride (LiCl; Sigma, L9650), sodium chloride (NaCl; Aladdin, C111533), and calcium chloride (CaCl_2_; Aladdin, C110768). The electronic conductors for the addition to PAM hydrogels were single-walled carbon nanotubes (SWCNTs, EFL-Conduct-SWCNTs), multi-walled carbon nanotubes (MWCNTs, EFL-Conduct-MWCNTs), and graphene (GO, EFL-Conduct-Graphene). Ecoflex (Smooth On, 00-30) was selected as the functional material of the dielectric elastomer of the flexible actuator.

### 2.2. Methods

When they are compared with general ion gels, the hydrogels based on salt electrolytes have the advantages of having high conductivity, a controllable cost, and a simple preparation, and have irreplaceable practical application value. In salt-based electrolyte hydrogels, the salt and the water each play a key role. The salt that acts as the electrolyte provides the conductive ions, and the water acts as a solvent to support the conductive particles. Therefore, the water retention capacity of the hydrogels is a fundamental property that affects their comprehensive properties. However, judging from the applications of hydrogels, their water retention capacity is poor, and they often lose too much water within a few hours, which greatly weakens their electrical conductivity. Encapsulation is a water retention method, but the operation of it is complicated and affects the application effect. Another method is to improve the water retention capacity of the hydrogels themselves by adjusting the type and concentration of salts. Solvated the hygroscopic salts can achieve the dual purpose of improving the water retention capacity and ionic conductivity of the hydrogels. However, due to the non-ideal nature of the electrolyte solution, the conductivity will decrease at higher salt concentrations, so it is necessary to maximize the retention of the water and ionic conductivity [27].

According to a modern electrolyte solution theory, the salts dissolve in water and ionize into cations and anions. At the same time, the ionized ions bond with water molecules to form hydrated ions. When it is compared with pure water, the ionic hydration effect in the salt solution usually changes its properties such as its volume, resistivity, freezing point, etc., especially in high concentration solutions [28]. Therefore, the water retention capacity and electrical conductivity of the hydrogels can be improved by appropriately selecting the dissolved salts and adjusting their concentrations [29]. We tried to introduce different types and concentrations of salts into the PAM hydrogels, and we studied their water retention capacity and electrical conductivity, aiming to find suitable salts and certain ratios to improve the performance of the hydrogels. Subsequently, we tried to introduce SWCNTs, MWCNTs, and GO electronic conductors to enhance the conductivity of the PAM hydrogels. In addition, we performed repeated tensile tests on the conductive hydrogel samples to ensure that they remained functional after repeated external stress. In order to test the performance of the PAM conductive hydrogels in practical applications, we made PAM conductive hydrogels with high water retention and good conductivity properties into flexible electrodes. Then, we applied these hydrogel electrodes to the dielectric elastomer flexible actuator, and then, we carried out the actuation experiment under the external electric field excitation condition.

### 2.3. Preparation of Experimental Hydrogel Samples

We prepared the deionized water as needed, the AM monomer (2.2 mol/L) and salt powder (LiCl: 2~6 mol/L, NaCl: 2~6 mol/L, CaCl_2_: 2~4 mol/L) were added to it for them to dissolve, then we used MBAA (0.06% the weight of AM) to make the AM monomer complete the cross-linking behavior. In addition to the AM monomer and salts, AP (0.17% the weight of AM) acted as an initiator and TEMED (0.25% the weight of AM) acted as a crosslinking accelerator. We poured the prepared hydrogel solutions into self-made acrylic molds and gel for 3 h in a temperature chamber of 50 °C [30]. The as-prepared hydrogel samples are referred to as PAM_LiCl(m), PAM_NaCl(m), and PAM_CaCl_2_(m), while m represents the initial dissolved salt concentration. Based on PAM_LiCl(m), the SWCNTs, MWCNTs, and GO were introduced to prepare new composite hydrogel samples. The as-prepared hydrogel samples are referred to as PAM_LiCl(m, S), PAM_LiCl(m, M), and PAM_LiCl(m, G).

### 2.4. Water Retention Capacity Testing Method

The hydrogel samples that were used for the water retention test were PAM, PAM_LiCl(2~6), PAM_NaCl(2~6), and PAM_CaCl_2_(2~4). In the preparation of the hydrogel samples, a cylindrical hydrogel sample with a size of φ40 mm × 5 mm was obtained after shaping it using a self-made acrylic mold, and it was continuously stored in an indoor environment with a stable temperature (25 °C) and relative humidity (60% RH). The hydrogel samples were placed on anti-adherent films (EFL-GRF-001) during the experiment to ensure that the changes in their dehydration shrinkage were not limited. During the experiment, the state of the hydrogel samples was recorded regularly for reference and comparison. To quantitatively evaluate the water loss of the hydrogels, their weight was recorded at certain times using an electronic scale, and the water loss at time = t is calculated by:

(1) $$\mathrm{Water}\;\mathrm{loss}=\frac{\mathrm{Weight}\;(\mathrm{hydrogel},\;\mathrm{time}=0)\;-\;\mathrm{Weight}\;(\mathrm{hydrogel},\;\mathrm{time}=t)}{\mathrm{Weight}\;(\mathrm{water},\;\mathrm{time}=0)}$$

where the weight (water, time = 0) is calculated by the amount of the raw materials that were used.

### 2.5. Conductivity Testing Method

The hydrogel samples for the conductivity testing were PAM_LiCl(2~6), PAM_NaCl(2~6), and PAM_CaCl_2_(2~4), and the hydrogel samples for the conductivity enhancement testing were PAM_LiCl(m, S), PAM_LiCl(m, M), and PAM_LiCl(m, G). After measuring the resistivity ρ of the hydrogel samples using a resistivity tester, it was converted according to Formula (2) to obtain the electrical conductivity σ. The electrical conductivity σ is calculated by:

(2) $$\sigma =\rho^{-1}$$

The resistivity ρ was measured using a four-probe resistivity tester (HELPASS, HPS2662) in cooperation with the resistivity test bench (HELPASS, HPS58001) and resistivity test probe (HELPASS, HPS58006/HPS58003).

### 2.6. System Reliability Tensile Testing Method

Two types of conductive hydrogel samples were selected for the system reliability tensile test. The PAM_LiCl(6) conductive hydrogel samples had two shapes which were a circular sample φ40 mm × 5 mm and a square sample 30 mm × 30 mm × 5 mm, respectively. We performed 500 repeated tensile tests on the two hydrogel samples using a tensile-compression testing machine. For the circular samples, the stretch limit was 3 times the diameter, and a 360° radial stretch around the circumference was performed. For the square samples, the stretch limit was 3 times the width. After the tensile test, the external morphology of the sample was observed, and then, the electrical conductivity test was performed on the tested sample to evaluate their system’s reliability.

### 2.7. Fabrication of Flexible Actuator Based on PAM Conductive Hydrogel Electrodes

The flexible actuator based on the PAM conductive hydrogel electrodes is similar to a sandwich structure as a whole, with there being a dielectric elastomer film in the middle and PAM conductive hydrogel electrodes on both sides of the dielectric elastomer film. We operated the experiment in strict accordance with the product instructions. The manufacturing process is as follows:

(1) Mix component A and component B of Ecoflex 00-30 at 1:1, centrifuge it at an acceleration of 1500× *g* for 10 min, and drain the internal bubbles to prevent them affecting the production effect;
(2) Pour the centrifuged mixture Ecoflex 00-30 material into a mold with a diameter of 60 mm for curing. The curing process lasts for 30 min in an oven at 60 °C. The dielectric elastomer layer with a diameter of 60 mm and a thickness of 1 mm will then be fabricated;
(3) Pre-stretch the prepared Ecoflex dielectric elastomer film and fix it with a circular acrylic fixing frame with an inner diameter of 80 mm;
(4) The surfaces of the dielectric elastomer will then be dried with nitrogen to enhance the adhesion of the hydrogels to the surfaces of the Ecoflex dielectric elastomer. Then, the pre-fabricated PAM conductive hydrogel electrodes will be closely bonded with the upper and lower sides of the pre-stretched dielectric elastomer film. The PAM conductive hydrogel electrodes on both sides will be used as the electrode lead out wire at an angle of 180° through the hydrogel thin wire, and they will be connected with the output end of the high-voltage power amplifier through the copper electrodes.

### 2.8. Actuation Experiment of Flexible Actuator

**Figure 1:**
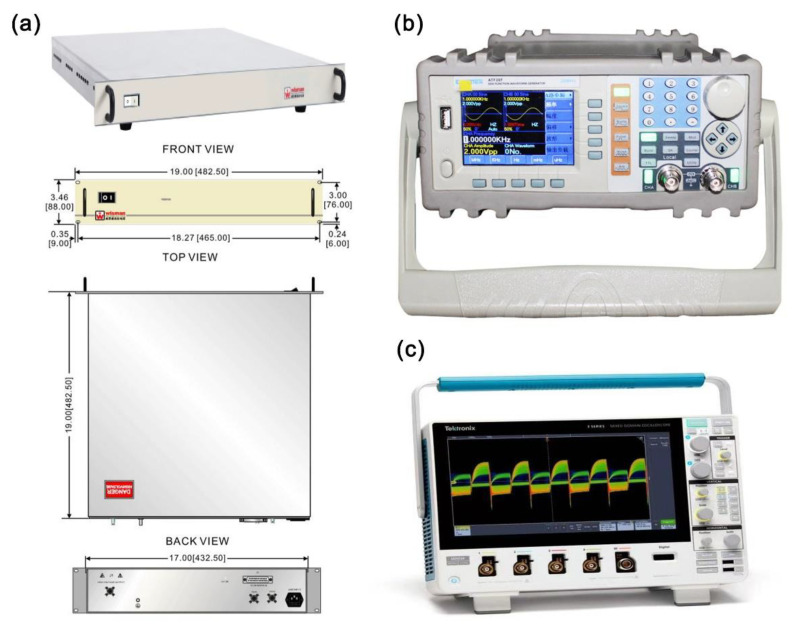
Main experimental equipment for the actuation mechanism experiment of flexible actuator based on PAM conductive hydrogel electrodes: (**a**) high-voltage power amplifier; (**b**) function signal generator; (**c**) digital oscilloscope.

Before conducting the actuation experiment of the flexible actuator based on the PAM conductive hydrogel electrodes, we prepared the corresponding experimental equipment and apparatus to ensure the successful implementation of the experiment. The required experimental equipment are shown in Table 1, and the appearance of them is shown in Figure 1.

## 3. Results and Discussion

### 3.1. Water Retention Capacity Test

**Figure 2:**
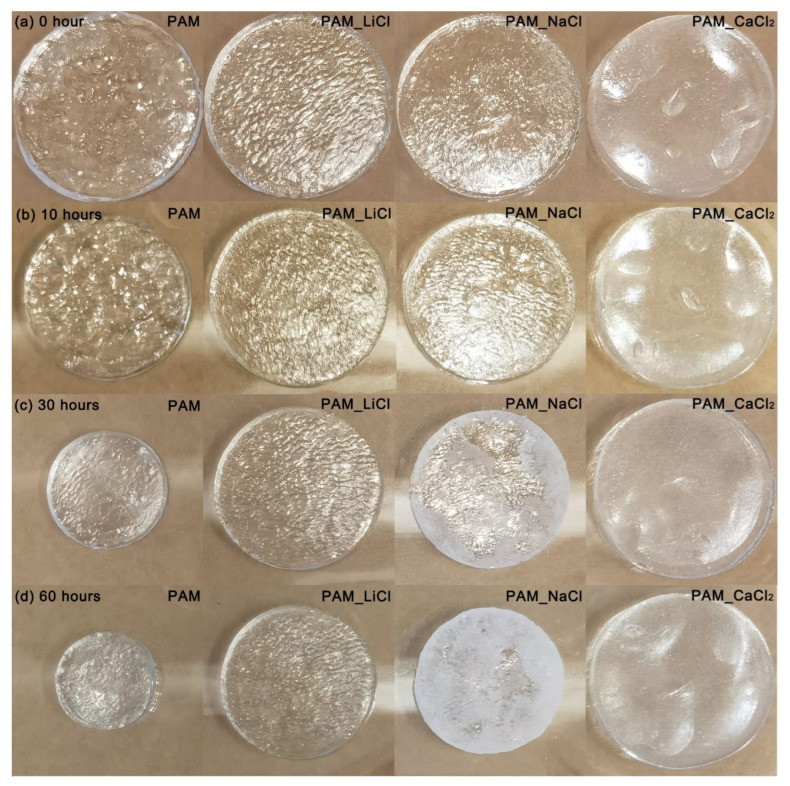
Photos of hydrogel samples kept in an environment at a temperature of 25 °C and relative humidity (RH) of 60% for different periods: (**a**) 0 h; (**b**) 10 h; (**c**) 30 h; (**d**) 60 h. The initial dissolved salt concentration of the hydrogel samples was 4 mol/L. The transparent hydrogel samples were put on anti-adherent films (EFL-GRF-001) in order not to constrain their shrinkage.

The size of the cylinder hydrogel samples were φ40 mm × 5 mm. We recorded the morphological changes of the hydrogel samples during this process using a camera as a qualitative reference. Figure 2 shows the photos of different hydrogel samples that were stored for different times in an environment at a temperature of 25 °C and a relative humidity (RH) of 60%. The initial dissolved salt concentrations of the selected hydrogel samples were all 4 mol/L.

All of the hydrogel samples were the same, and a certain degree of shrinkage could be seen after 10 h (Figure 2b). This indicates that some of the water in the hydrogel sample had evaporated. Among them, the PAM hydrogel sample showed the largest change, the PAM_LiCl hydrogel sample showed the smallest change, and the PAM_CaCl_2_ hydrogel sample had no visible morphological changes. After about 30 h, as shown in Figure 2c, the unsalted PAM hydrogel sample had shrunk substantially, and it had become stiffer. The PAM_NaCl hydrogel sample had precipitated some NaCl salts, indicating that it had lost a lot of water. However, the PAM_LiCl hydrogel sample just continued to shrink, but the shrinkage rate was significantly lower than it had been before, indicating that the evaporation rate of water became slower. It is worth noticing that there is almost no morphological change in the PAM_CaCl_2_ hydrogel sample during this process. After about 60 h, as shown in Figure 2d, the unsalted PAM hydrogel sample had hardened, and it had almost completely lost its water. The PAM_NaCl hydrogel sample almost turned into a round salt block. The PAM_LiCl hydrogel sample continued to shrink at a lower shrinkage rate, which indicates that the rate of the evaporation of its water became slower. From the next 60 h to 110 h, all of the hydrogel samples did not undergo any morphological changes, indicating that they all had reached a stable state of water loss.

**Figure 3:**
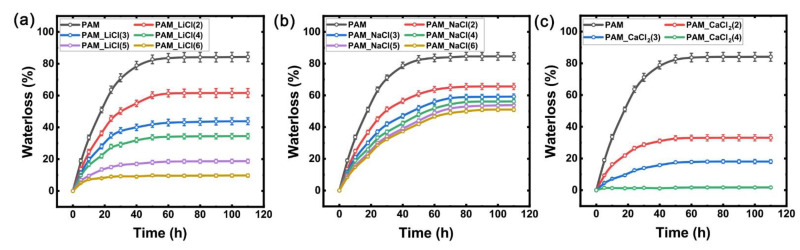
Evolutions of water loss with time for different hydrogel samples kept in environment with temperature of 25 °C and relative humidity (RH) of 60%: (**a**) PAM_LiCl(m); (**b**) PAM_NaCl(m); (**c**) PAM_CaCl_2_(m).

During the experiment, the water loss of all of the hydrogel samples was quantitatively recorded according to Formula (1). Figure 3 quantitatively shows the water loss of the PAM_LiCl(m), PAM_NaCl(m), and PAM_CaCl_2_(m) samples (PAM hydrogel samples containing different concentrations of LiCl, NaCl, and CaCl_2_) in a chamber at a temperature of 25 °C and a relative humidity (RH) of 60%. According to the trend of the curves in Figure 2, it can be seen that different hydrogel samples showed similar water loss characteristics. Over time, the water loss gradually intensified, and it reached a stable state after about 60 h. The steady-state water loss of unsalted PAM hydrogel sample reaches 85%. For the PAM_LiCl hydrogel sample (Figure 3a), the steady-state water loss rate decreased from 62% to 10% as the initial concentration of LiCl increased from 2 mol/L to 6 mol/L. For the PAM_NaCl (Figure 3b), the steady-state water loss rate decreased from 65% to 50% as the initial concentration of NaCl increased from 2 mol/L to 6 mol/L. Additionally, for the PAM_CaCl_2_ hydrogel sample, when the initial concentration of CaCl_2_ increased from 2 mol/L to 3 mol/L, the steady-state water loss rate decreased from 33% to 18% (Figure 3c). When the concentration increased to 4 mol/L, almost no water loss occurred. Obviously, the added salts all played a role in improving the water retention capacity of the hydrogels. For the hydrogels containing the same salt, with the increase in the salt concentration, the water loss rate decreased, the steady-state water loss rate decreased, and the water retention capacity became stronger. For the hydrogels containing different kinds of salts, the differences in water loss rate and steady-state water loss rate were mainly determined by the type of salts. However, the specific relationship between the water loss, salt type, and salt concentration needs to be explored and analyzed through more experiments. Compared with the PAM_NaCl hydrogel sample with severe water loss, the PAM_LiCl hydrogel sample and PAM_CaCl_2_ hydrogel sample had a stronger water retention capacity, especially PAM_CaCl_2_(4), which could even achieve almost no water loss. 

In the experiment, the hydrogels added with CaCl_2_ and LiCl exhibited better water retention capacities than the NaCl one did, which is essentially due to the difference in the properties of the salts themselves. CaCl_2_ is a colorless cubic crystal with strong hygroscopicity, which is easily deliquescent and water-soluble when it is exposed to air [31]. CaCl_2_ is a commonly used desiccant, and the moisture absorption of the industrial-grade pure CaCl_2_ (purity 70%) can reach 100% of its own mass. Additionally, anhydrous CaCl_2_ (purity 95%) can absorb moisture up to 150% of its own mass [32]. LiCl is an inorganic substance with white crystals, it is deliquescent and easily soluble in water, and it is a commonly used desiccant [33]. The radius of Li^+^ is too small (the radius has a non-negligible effect on the properties of the material), so its polarizing force is very large (it can be determined by the ionic potential the ionic potential is the charge-to-the radius ratio), and the attraction to the electrons is stronger. The oxygen ions (O^2−^) in water occupy a large number of electrons, so the lithium ions have a strong ability to attract water [34]. NaCl is an inorganic ionic compound in the form of colorless cubic crystals or a fine crystalline powder. Its source is mainly seawater, which is commonly used in industrial production, medical treatment, and in life. NaCl is more stable and easy to store, so its hygroscopic performance is not as good as that of CaCl_2_ and LiCl. This also leads to the phenomenon of more water loss in the hydrogels that are added with NaCl.

The different water retention capacity that is exhibited by these hydrogel samples is caused by differences in the ionic hydration of the different salts. In the hydrogels, the water molecules that are bound to ions must break their bonds to evaporate, while the free water molecules can evaporate naturally. The higher that the degree of the ionic hydration of the salt is, the stronger the bond is between the cation/anion and the water molecule [35]. Therefore, the more difficult it is for the water molecules to evaporate and become lost, then the stronger the water retention capacity is. The experimental results show that the degree of ionic hydration of the salts is controlled by the salt species and salt concentration.

In this section of the experiments on the water retention performance of the hydrogels, only the surface relationship between the water loss of the hydrogels and the type and content of salts was analyzed in a simple way. Essentially, the changes to the mechanical properties exhibited by the hydrogels are the most reasonable objects of study. Therefore, it is more intuitive and clear to investigate the mechanical strength of the hydrogels as a function of the salt and water content as well as a function of water loss, which is also the direction in which we want to carry out research in the future.

### 3.2. Conductivity Test

The type and concentration of the salts not only affects the water retention capacity of the hydrogels, but also their electrical conductivity. This section discusses the conducted conductivity tests based on these hydrogel samples to determine the effect of the salt type and concentration on the conductivity of the hydrogels. After the hydrogel samples reached a stable state of water loss, the resistivity ρ was measured using a four-probe resistivity tester, and then, the conductivity σ was obtained according to Formula (2).

**Figure 4:**
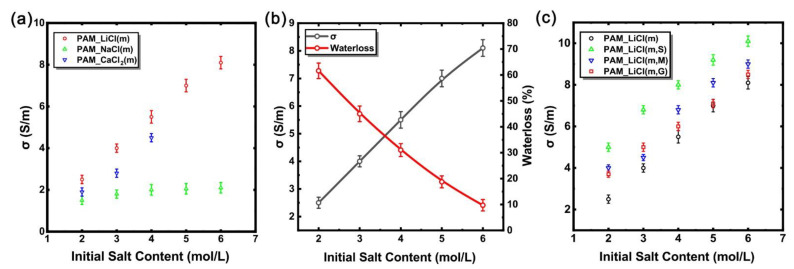
(**a**) The conductivity of steady-state hydrogel samples containing different salts with varied concentration when kept in a chamber at a temperature of 25 °C and a relative humidity of 60% RH; (**b**) changes in steady-state water loss rate and conductivity of PAM_LiCl hydrogels with initial dissolved concentration; (**c**) conductivity of steady-state PAM_LiCl(m, S), PAM_LiCl(m, M), and PAM_LiCl(m, G).

Figure 4a shows the steady-state conductivity of the different hydrogel samples that were stored in a chamber at a temperature of 25 °C and a relative humidity (RH) of 60%. The results show that, for the hydrogels containing the same kind of salts, when the initial dissolved salt concentration increased, its electrical conductivity increased with the increase in the initial dissolved salt concentration. The steady-state conductivity of the PAM_LiCl(6) sample is the highest; it is about 8 S/m. In contrast, the steady-state conductivity of the PAM_NaCl(6) sample is about 2 S/m, while the steady-state conductivity of the PAM_CaCl_2_(4) sample is about 4.5 S/m. The results show that the electrical conductivity of PAM_LiCl hydrogel sample is much higher than those of PAM_NaCl hydrogel sample and PAM_CaCl_2_ hydrogel sample. LiCl can improve the water retention and ionic conductivity of the hydrogels. Within a certain range, the water retention capacity and conductivity of the hydrogels will increase with the increase in the initial salt concentration. Figure 4b summarizes the experimental results of LiCl on improving the water retention capacity and conductivity of the PAM hydrogels, thus confirming the advantages of LiCl in improving the comprehensive performance of the PAM hydrogels and identifying it as a main research object in the follow-up experiments.

The traditional conductive materials are mostly electronic conductors, and the salt-containing ion-conducting hydrogels are mainly ion-conductive. Although this hydrogel has advantages in it being transparent and stretchable, it sometimes has a large gap between the electronic conductors during electrical conductivity, which makes it difficult for it to meet the application requirements. Based on the above conductivity test results, the PAM_LiCl(m) hydrogels with strong conductivity and water retention capacity properties were selected for the conductivity enhancement experiment. We tried to add a certain amount of SWCNTs, MWCNTs, and GO electronic conductors to enhance their conductive properties, thereby constructing novel composite conductive hydrogels. This is a new attempt. Due to the limitation of the objective conditions, the experiment of this attempt has not been carried out more deeply, but we hope that this can provide people with a new idea.

Figure 4c shows the steady-state conductivity of the hydrogel samples that were added with different electronic conductors. At the same concentration, the hydrogel samples with electronic conductors do have higher conductivity values. Among the three added electronic conductors, the SWCNTs have the best effect on the enhancement of the conductivity, enhancing the electrical conductivity of PAM_LiCl(6) from about 8 S/m to about 10 S/m. In contrast, the effects of MWCNTs and GO are weak, only enhancing the electrical conductivity of PAM_LiCl(6) to about 9 S/m and 8.5 S/m, respectively. The experimental results show that the addition of these three electronic conductors can indeed enhance the conductivity of the hydrogel, but the enhancement effect is different, and the SWCNTs have the best conductivity enhancement effect. It should be noted that properties such as the transparency and stretchability of the hydrogel itself are affected to varying degrees after adding these electronic conductors. Therefore, it was necessary to consider whether to add such electronic conductors and the amount that should be added according to the actual situation. In addition, the way of adding them may affect the effect, and in this experiment we only used a simple mixing method, so the effect was limited.

**Figure 5:**
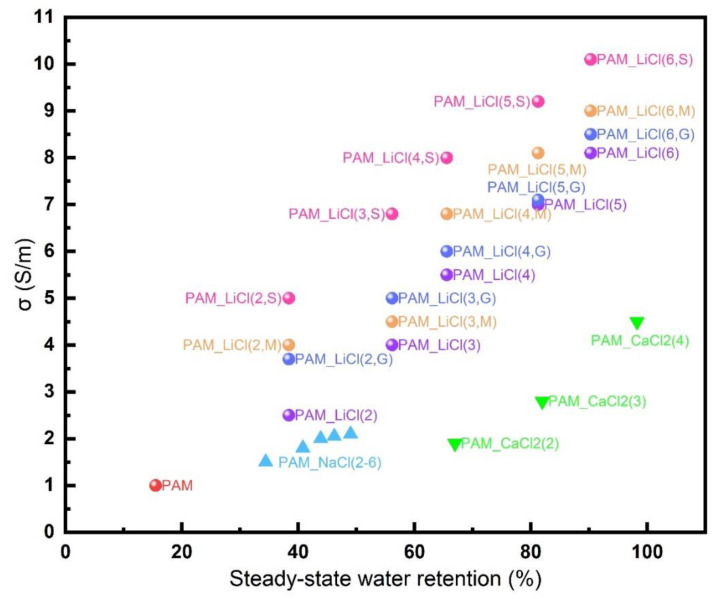
An Ashby diagram of hydrogel samples involved in all of the experiments.

Figure 5 is an Ashby diagram containing all of the hydrogel samples involved in this experiment. The parameter information of all of the hydrogel samples with different ratios can be clearly obtained in Figure 5. PAM_LiCl (6, S) is located in the upper right corner of Figure 5, representing that it has the best water retention and conductivity of all of the experimental samples. PAM is located at the lower left corner, which means that the water retention and conductivity of the pure PAM hydrogel samples without any additional ingredients are the worst among all of the experimental samples. The other PAM hydrogel samples with different ingredients and additional materials have different locations in the middle of Figure 5.

The experiments that are in this chapter only observed the macroscopic phenomena, and the differences in these macroscopic phenomena are essentially due to changes in the microstructure. When the experimental conditions were improved, X-ray diffraction (XRD), Raman spectroscopy, infrared radiation (IR) infrared spectroscopy, Fourier transform infrared spectroscopy (FTIR) and other techniques should be used to observe and analyze the internal structure, morphological characteristics, bonding strength, etc., of the hydrogels. Only in this way can the macroscopic phenomena be explained fundamentally and using deep scientific principles.

### 3.3. System Reliability Tensile Test

**Figure 6:**
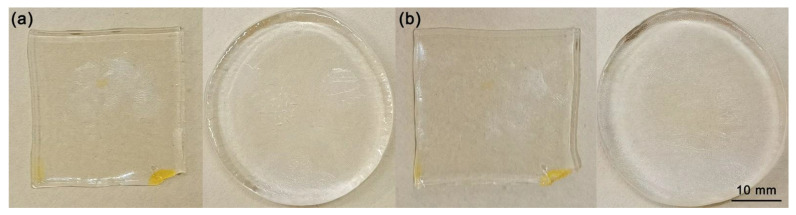
(**a**) Test samples in initial state; (**b**) test samples after repeated stretching 500 times.

In order to ensure that the conductive hydrogel samples can still maintain a normal performance after undergoing repeated external stretching, the system reliability tensile test is carried out in this section. In this section, two kinds of PAM_LiCl(6) conductive hydrogel samples were selected, as shown in Figure 6a; the round sample was φ40 mm × 5 mm, and the square sample was 30 mm × 30 mm × 5 mm. In the experiment, we performed 500 repeated tensile tests on the two hydrogel samples. For the circular samples, we set the stretch limit at three times the diameter and performed a 360° radial stretch along the circumference. For the square samples, we set the stretch limit to be three times the width. The morphological characteristics of the two samples did not change significantly after 500 repetitions of stretching, as shown in Figure 6b. After conducting a conductivity test on the samples after the system reliability tensile test, their conductivity was basically the same as the conductivity test results in Section 2.2. The conductivity of the round sample is about 7.6 S/m, and the conductivity of the square sample is about 7.8 S/m. Experiments show that the system can still maintain normal morphological characteristics and performance after being subjected to repeated external stresses.

After 500 repeated tensile tests, the system can still maintain normal morphological characteristics and properties. In order to further test the reliability of the system, we increased the repeated stretching times to 1000 times, 3000 times, and 5000 times, respectively. Based on the principle of control variables, the conductive hydrogel samples used in the supplementary experiments maintained the same shape characteristics. Three round samples were φ40 mm × 5 mm, and three square samples were 30 mm × 30 mm × 5 mm. In the process of repeated tensile testing, for the round samples, we set the tensile limit to three times the diameter, and we carried out 360 ° radial tensile along the circumference. For the square samples, we set the stretching limit to three times the width. A total of six conductive hydrogel samples with two shapes have undergone 1000, 3000, and 5000 repeated tensile tests, respectively. After the tensile tests, the morphological characteristics of all of the conductive hydrogel samples did not change significantly. After the conduct conductivity test on all of the samples, the conductivity was basically stable. The conductivity of the round samples fluctuates at around 7.4 S/m, and that of square samples fluctuates at around 7.5 S/m. Further repeated tensile tests proved that the PAM conductive hydrogels could still maintain normal morphological characteristics and performance and meet the stability requirements under general conditions after being subjected to repeated external stresses.

### 3.4. Actuation Experiment of Flexible Actuator Based on PAM Conductive Hydrogel Electrodes

The flexible actuator based on the PAM conductive hydrogel electrodes is highly stretchable. As shown in Figure 6, in the two sides of the flexible actuator were pre-fabricated circular PAM conductive hydrogel electrodes, φ30 mm, with a thickness of about 1 mm, extending 180 ° through the hydrogel thin wire to the acrylic fixed frame to apply external voltage. The intermediate layer was a cured Ecoflex dielectric elastomer film, φ60 mm, with a thickness of 1 mm. The intermediate Ecoflex dielectric elastomer layer, which has been pretensioned to a certain extent, was fixed with a circular acrylic rigid frame with an inner diameter of 80 mm. The upper and lower sides of the flexible actuator were covered with circular conductive hydrogel electrodes with a small area, which were led to the acrylic frame through the hydrogel thin wire, and then, connected to the high-voltage power output terminal through the copper electrodes. 

**Figure 7:**
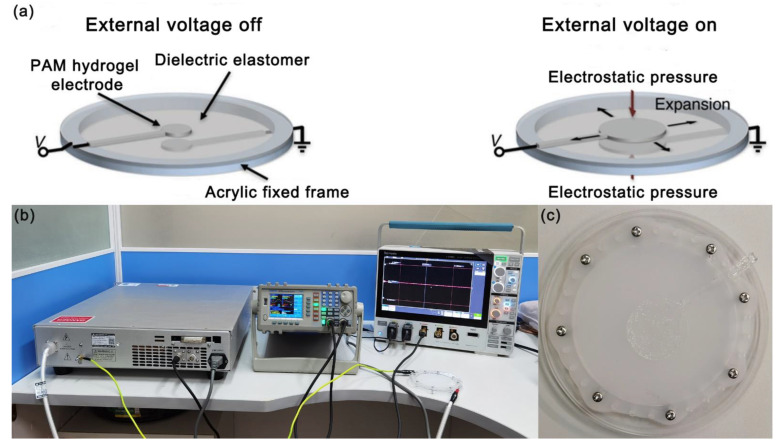
(**a**) Schematic diagram of actuating mechanism of flexible actuator based on PAM conductive hydrogel electrodes; (**b**) the whole experimental device for the verification experiment of the actuating mechanism of the flexible actuator; (**c**) upper view of flexible actuator based on PAM conductive hydrogel electrodes.

As shown in Figure 7a, when a voltage was applied between the PAM conductive hydrogel electrodes on both sides, ions with different charge polarities gathered on two surfaces of the dielectric. The interfaces with opposite charges attract each other, resulting in the decrease in the thickness and the expansion of the area of the intermediate layer dielectric elastomer. The flexible actuator based on the PAM conductive hydrogel electrodes can be divided into three parts as a whole: the upper hydrogel electrode (with an external electrode attached), dielectric elastomer, and the lower hydrogel electrode (with an external electrode attached). The upper view is shown in Figure 7c. The external electrodes are copper electrodes with a good conductivity property. They are directly connected to the output channel of the external voltage. Figure 6b is the overall experimental device for the actuation mechanism verification experiment of the flexible actuator based on the PAM conductive hydrogel electrodes. The function signal generator provided an adjustable signal input. The high-voltage power amplifier amplified the function signal to the kilovolt level voltage that was required by the experiment (the input signal 0–10 V corresponds to the output voltage 0–10 kV). The hybrid domain digital oscilloscope was used to monitor the output signal of the high-voltage power amplifier. When the external voltage was turned on, the middle Ecoflex dielectric elastomer layer expanded in the area direction. At this time, the area of the conductive hydrogel electrodes closely fitted with the Ecoflex dielectric elastomer layer that expanded with it, showing an actuating effect.

**Figure 8:**
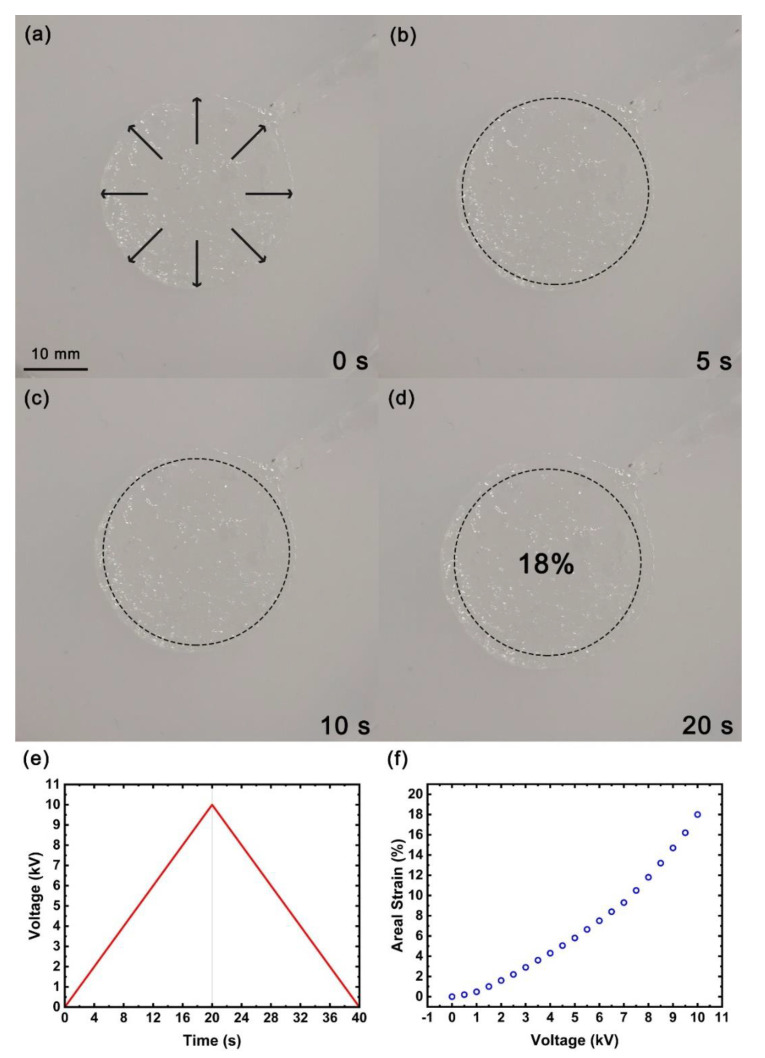
Flexible actuator based on PAM conductive hydrogel electrodes. (**a**–**d**) When an external voltage of triangular waveform with a period of 40 s is applied, the area of the flexible actuator expands over time; (**e**) triangular waveform external voltage with a period of 40 s; (**f**) the area strain measured at 20 s after the voltage was applied.

First, we applied a triangular wave voltage signal with a period of 40 s to the flexible actuator based on the PAM conductive hydrogel electrodes, as shown in Figure 8e. The rising area of the triangular waveform voltage signal slowly and linearly rose from 0 V to 10 kV within 20 s, and the falling area slowly and linearly dropped from 10 kV to 0 V within 20 s. During this process, we used the camera to record the changes of the PAM conductive hydrogel electrodes on the flexible actuator, as shown in Figure 8a–d. According to Formula (3), the area strain of the circular PAM conductive hydrogel electrodes changed with the applied voltage, as shown in Figure 8f.

(3) $$\mathrm{Areal}\;\mathrm{Strain}=\frac{\mathrm{Action}\;\mathrm{area}-\mathrm{Static}\;\mathrm{area}}{\mathrm{Static}\;\mathrm{area}}\times 100\%$$

As shown in Figure 8a–d, when the external applied voltage was in the rising region of the triangular wave, the circular PAM conductive hydrogel electrodes slowly expanded with the increase in the voltage. It can be seen from Figure 8e,f that when the applied voltage reached 10 kV, the area strain of the circular PAM conductive hydrogel electrodes reached a maximum of about 18%. When the applied voltage was then slowly removed, the circular hydrogel electrode slowly shrank as the voltage decreased. When one is trying to increase the frequency of the voltage signal, the expansion and contraction will show a certain rhythmic expansion and contraction effect will be similar to the beating of a heart. However, with the increase in voltage signal frequency, the maximum area strain will gradually decrease due to the response characteristics and viscosity effect of the material itself. If the shape of the hydrogel electrode is changed to a heart shape, it will look like the beating effect of a heart. There should be some correlation between the area strain of the PAM conductive hydrogel electrodes and the frequency of the voltage signal, which requires further experimental observations.

**Figure 9:**
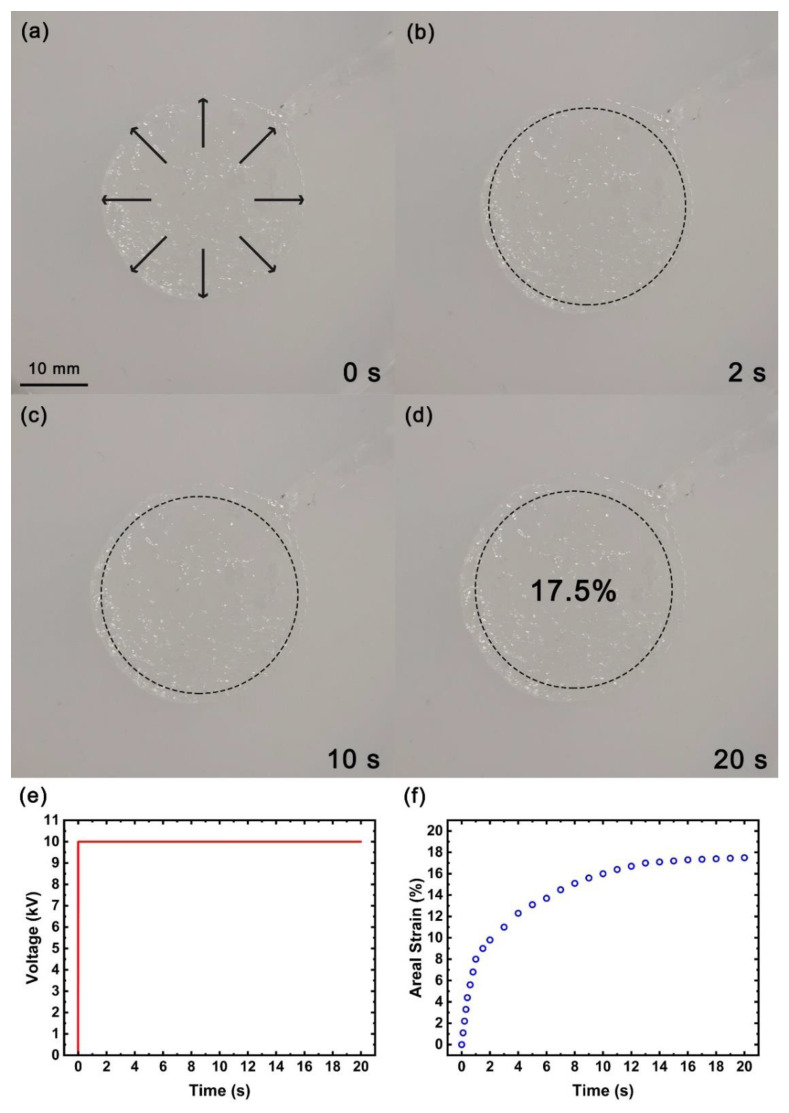
Flexible actuator based on PAM conductive hydrogel electrodes. (**a**–**d**) When a voltage was suddenly applied and subsequently held constant, the actuator expanded in the area over time; (**e**) waveform of suddenly applied external voltage; (**f**) the area strain measured at 20 s after the voltage was applied.

Then, we tried to apply a certain voltage to the flexible actuator based on the PAM conductive hydrogel electrodes suddenly and keep it constant. The voltage signal is shown in Figure 9e. At the beginning, the external voltage of 10 kV was applied, and it was kept constant. During this process, we used the camera to record the changes of the PAM conductive hydrogel electrodes on the flexible actuator, as shown in Figure 9a–d. We recorded the change to the area strain of circular hydrogel electrodes over time according to Formula (3), as shown in Figure 9f. The flexible actuator expanded suddenly over a very short time at the beginning, and then, it slowly expand over time, and the expansion rate decreased after about 20 s. The maximum area strain of the PAM conductive hydrogel electrodes was about 17.5% at 20 s.

In the above experiments, we used a relatively thick material layer to properly simplify the material processing when we were making flexible actuator. In many practical applications, people may want to reduce the thickness proportionally to reduce the applied voltage to ensure the safety and reliability of the system [36]. Although the flexible actuator based on the PAM conductive hydrogel electrodes in this section achieved the actuating effect under the excitation of a high-voltage electric field; the maximum response amplitude was about 18%, which is much smaller than those of many practical applications. By summarizing the experimental process and analyzing the problems, the selection of the fabrication technology and materials may be one of the factors affecting the experimental results. For the specific impact relationship, further experimental exploration and solutions are needed to achieve the expected large deformation and approximate the dynamic drive of this flexible actuator, which can actually meet the requirements of the actual application level.

## 4. Conclusions

In summary, the paper investigated the effects of the type and concentration of electrolyte salts on the water retention capacity and conductivity of PAM hydrogels. In terms of improving the water retention capacity of the hydrogels, LiCl and CaCl_2_ have excellent effects, and the water retention increased with the increase in the initial dissolved salt concentration. When the initial dissolved salt concentration of LiCl was 6 mol/L, more than 90% of the water in PAM_LiCl(6) was retained in the experimental environment. Furthermore, PAM_LiCl(6) also exhibits an excellent conductivity property (a high electrical conductivity of about 8 S/m). We also found that the ion-electronic composite conductive hydrogels that were obtained by adding electronic conductors such as SWCNTs, MWCNTs, and GO have better conductivity properties. In particular, the electrical conductivity of the PAM_LiCl(6) with the addition of the SWCNTs was improved from 8 S/m to about 10 S/m. In addition, after 500 repeated tensile tests, the conductive hydrogel samples could still maintain their original morphological characteristics, with a certain degree of system reliability. In addition, we applies these PAM conductive hydrogels with high water retention and good conductivity to an electric response flexible actuator. The flexible actuator itself was highly stretchable and could achieve a maximum area strain of 18% under an external voltage of 10 kV. These findings and new application attempts will contribute to the development of a new method for preparing composite conductive hydrogels with high water retention and excellent conductivity, thereby expanding the application fields of hydrogels.

## Figures and Tables

**Table 1:** Main experimental equipment and apparatus required for the actuation mechanism experiment of flexible actuator based on PAM conductive hydrogel electrodes.

Serial No	Name	Brand	Model	Number
1	Function signal generator	GRATTEN	ATF20F	1
2	High-voltage power amplifier	Wisman	AMR10R20	1
3	Mixed domain digital oscilloscope	Tektronix	MDO34	1

## Data Availability

Not applicable.
